# A brief review of recent developments in the designs that prevent bio-fouling on silicon and silicon-based materials

**DOI:** 10.1186/s13065-017-0246-8

**Published:** 2017-02-20

**Authors:** Xiaoning Zhang, DaShan Brodus, Valerie Hollimon, Hongmei Hu

**Affiliations:** 1 Department of Mathematics, Sciences and Technology, Paine College, 1235 Fifteenth Street, Augusta, GA 30901 USA; 2 Key Laboratory of Mariculture and Enhancement of Zhejiang Province, Marine Fishery Institute of Zhejiang Province, Zhoushan, 316021 China

**Keywords:** Silicon, Silicon-based materials, Antifouling, Surface modification, Biomimetic

## Abstract

Silicon and silicon-based materials are essential to our daily life. They are widely used in healthcare and manufacturing. However, silicon and silicon-based materials are susceptible to bio-fouling, which is of great concern in numerous applications. To date, interdisciplinary research in surface science, polymer science, biology, and engineering has led to the implementation of antifouling strategies for silicon-based materials. However, a review to discuss those antifouling strategies for silicon-based materials is lacking. In this article, we summarized two major approaches involving the functionalization of silicon and silicon-based materials with molecules exhibiting antifouling properties, and the fabrication of silicon-based materials with nano- or micro-structures. Both approaches lead to a significant reduction in bio-fouling. We critically reviewed the designs that prevent fouling due to proteins, bacteria, and marine organisms on silicon and silicon-based materials.

**Graphical abstract:**
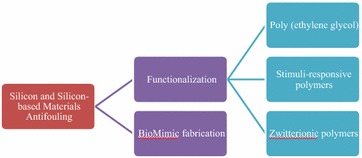
Strategies used in the designs that prevent bio-fouling on silicon and silicon-based materials.

Strategies used in the designs that prevent bio-fouling on silicon and silicon-based materials.

## Background

Silicon and silicon-based materials are integral parts of our daily life. Silicon has widespread applications in healthcare and manufacturing due to its unique material properties, including high flexibility, low biological activity, ease of fabrication, and chemical and thermal stability [1–4]. In addition, silicone based materials, such as silicone elastomer, is also the basic constituent of tubing, microfluidic system, catheters, drains, shunts, joint implants, silastic mammary prostheses, and contact lenses [5]. Silicon-based materials, such as Si_x_N_4_, have excellent fracture toughness and are chemically inert. Therefore, silicon is often used as an insulator and chemical barrier in manufacturing integrated circuits [6].

Unfortunately, silicon and silicon-based materials are susceptible to bio-fouling, which is the tendency of microbes, cells, and bacteria to physically adsorb to surfaces. This leads to deterioration of surface engineering in addition to infectious contamination [7], which are significant concerned [8]. For example, in the USA, about 40% of all bacterial infections occurring in hospitals are found to be catheter-associated urinary tract infection (CAUTI), which considerably increases the healthcare costs, the length of stay in the hospital and the antibiotic use [9–11].

Bio-fouling can also result in the growth of marine organisms on ship hulls, which is a major expense factor in naval industries. Based on the World Shipping Council’s report, fuel can represent as much as 50% of a ship’s total operating costs. Because the fouling causes a drag force on the ship and subsequent decreased fuel efficiency, hull fouling can increase fuel consumption, cost, and carbon dioxide emissions by as much as 40% [12]. In addition, the metabolic activity of the attached organisms can cause localized corrosion [13]. In one solution to this problem, polydimethylsiloxane (PDMS), a low energy silicone material, was used as a nontoxic alternative to conventional biocide paints.

**Fig. 1:**
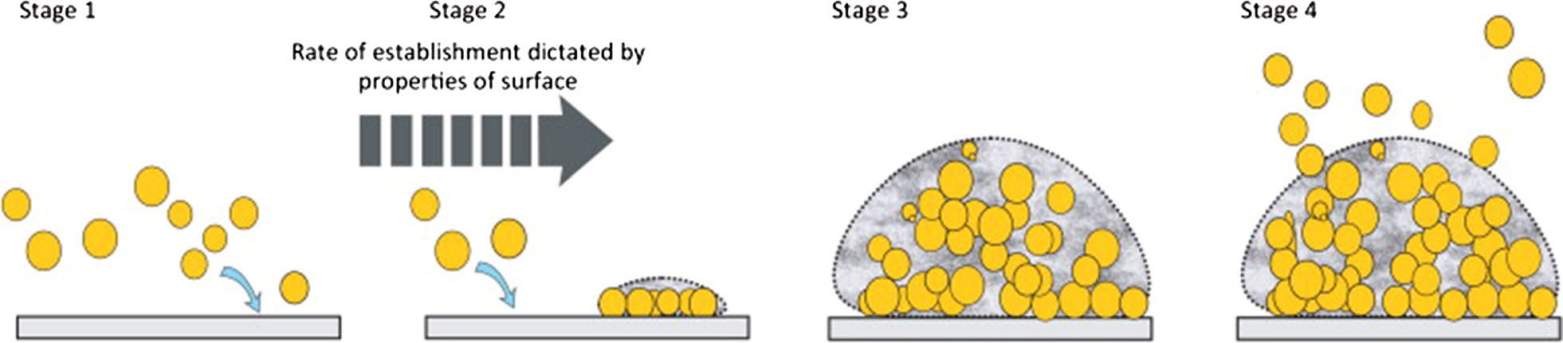
The four stages of biofilm formation [16] reproduced with permission from Elsevier

According to previous studies, bio-fouling develops in four stages [14]. In stage 1, bio-organisms encounter a surface and form a confluent layer. In stage 2, the bio-organisms multiply locally and then assemble to form a “microcolony”. The third stage of this process involves the formation of macrocolonies from the embedded microcolonies. Finally, once a mature biofilm is formed, bio-organisms exit and re-enter the biofilm structure as the last stage (Fig. 1).

Significant efforts have been dedicated to modifying silicon and silicon-based materials to improve their anti-fouling performance. The objective was to change the properties of the materials or the immobilized molecules in ways that either limit foulant accumulation, or provide ways to remove attached foulants after adsorption saturation, such that the fouled materials can be regenerated. However, reviews of surface modification of silicon and silicon-based materials for their antifouling and antibacterial properties are lacking. Therefore, in this article, an overview of the recent work in silicon and silicon-based materials modification for antifouling purposes will be presented. We are interested in silicon-based materials such as silicone elastomer because they are appropriate to be used for medical applications.

## Functionalization of silicon and silicon-based substrates with antifouling molecules

### Poly (ethylene glycol) and its derivatives

As the most commonly-used materials for fouling resistance, poly (ethylene glycol) (PEG) and its derivatives are widely used to engineer the surface of silicon [15–17]. Their modification enables silicon to be hydrophilic, nontoxic, and biocompatible. The hydration layer surrounding the ethylene oxide chain is the reason PEG has demonstrated the ability to repel fouling materials [18]. However, the ethylene oxide chains are, over time, auto-oxidized in aqueous solutions, resulting in cleavage of ethylene oxide units and formation of aldehyde-terminated chains. Therefore, there are limits to its long-term application (more than 14 days). In addition, the formed aldehyde moieties may react with fouling materials, such as protein, resulting in a declination of the repellent nature of the PEG coatings [19]. Furthermore, PEG loses its protein resistance at 37 °C and above, while 37 °C is a critical temperature for many biomedical applications. It is known that repulsive ethylene oxide (EO)-protein interactions are essential to the anti-fouling efficiency of PEG. Higher temperatures (37 °C and above) can cause EO monomers to alternate their configurations and affect EO-protein interactions, resulting in more protein adsorption on the surface [20].

**Fig. 2:**
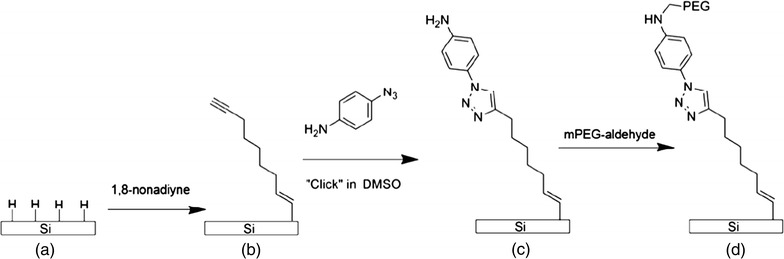
Schematic diagram of grafting PEG on silicon surface via click chemistry [24] reproduced with permission from ACS Publications

Recently, different surface modification methods have been implemented to graft PEG molecules onto silicon and silicon-based substrates. One of these includes self-assembly, in which PEG molecules are grafted on the silicon wafers or glass slides via silanization, which is achieved by leaving silicon slides or glass slides overnight in a solution of PEG in dry toluene [21]. Research groups from Germany and Australia reported a stepwise method for the construction of PEG layers onto a silicon surface via click chemistry [22]. In their work, acetylene-terminated alkyl monolayers were first attached to non-oxidized crystalline silicon surfaces through a hydrosilylation reaction. The acetylene-terminated surfaces were then functionalized via a copper-catalyzed azide-alkyne cycloaddition reaction to generate an amine-terminated layer. Eventually, a PEG layer with high graft density was conjugated with an amine-terminated substrate via an N–C bond (Fig. 2). The antifouling properties of this chemical-modified surface were investigated by testing adsorption of human serum albumin (HSA) and lysozyme (Lys). However, their results indicate that the surface is fully antifouling to large proteins, such as HSA, but does not completely repel the low-molecular weight protein, such as Lys. The study of protein adsorption on the surface is important, as in the clinical setting blood clots and subsequent thromboses may be more likely to develop with use of medical devices and implants.

In a report published in *Langmuir* [23], the copolymer, poly(TMSMA-r-PEGMA), which is comprised of an “anchor part” (trimethoxysilane) and a “function part” (PEG), was synthesized by a radical polymerization reaction. Then polymeric self-assembled monolayers (PSAMS) of poly(TMSMA-PEGMA) on Si/SiO_2_ or glass substrates were prepared by immersing the substrate in a methanol solution of poly(TMSMA-PEGMA) at ambient temperature. Then the polymer-coated Si/SiO_2_ or glass substrates were immersed in insulin, lysozyme, and fibrinogen solution to evaluate their protein resistance characteristics. The results demonstrated that the polymer-coated Si/SiO_2_ or glass substrates have a great reduction in nonspecific protein adsorption compared to the unmodified substrates.

**Fig. 3:**
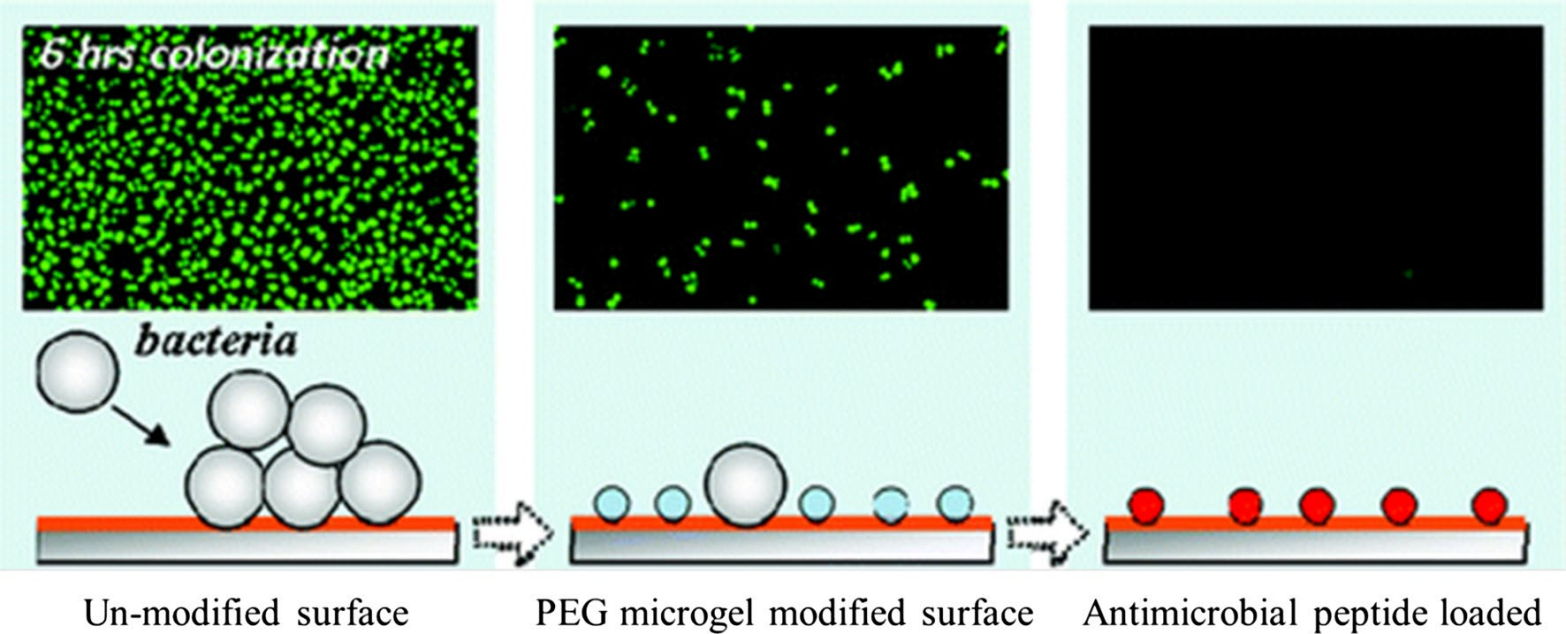
Microgels of PEG were deposited onto silicon substrates to inhibit bacterial colonization [31] reproduced with permission from ACS Publications

PEG-based materials also resist adhesion of many bacteria [24–29]. In research from Libera et al., PEG-based microgels were deposited on silicon substrates [30]. Because the average spacing between microgels on silicon substrates is approximately the same micrometer size of the bacterium itself, the bacterium experiences surface repulsiveness. In addition, the microgel modified-surfaces exhibit a reduced susceptibility to *S. epidermidis*, bacteria commonly implicated in biomaterial-associated infection. In further study, a cationic antimicrobial peptide (L5) was loaded onto the surface-bound microgels, and microgels serve as reservoirs for antimicrobial delivery. The additional loading of L5 into PEG-based microgels amplified the resistance of the microgel-modified surfaces to *S. epidermidis* colonization (Fig. 3). The major disadvantage associated with release coatings based on antimicrobial components is the depletion of the active species, which leads to a loss in the antimicrobial activity of the coatings with time. This problem may be addressed by using reloadable coatings. Another option is to use slow releasing coatings that release the biocide over longer periods of time [31, 32].

In another study, Voo and co-workers coated thiol-functionalized silicone rubber, which is a commonly used catheter material, with modified PEG molecules through a Michael addition reaction. The antibacterial and antifouling properties of the polymer-modified surface against both Gram-positive *Staphylococcus aureus,* which is methicillin-resistant and a major cause of the infection, and Gram-negative *Escherichia coli* were examined. Their results shown that the coating can prevent *S. aureus* and *E. coli* biofilm formation over 14 days incubation. Comparing to a high number of *S. aureus* and *E. coli* adhered onto the pristine surface after one day incubation, this coating demonstrates potential for use as antifouling coating to prevent catheter-associated infections [33].

Yang’s team created PEG nonfouling anti-microbiao hydrogels, which can be applied onto catheters or other implants as a coating to prevent infections. This PEG hydrogel was fabricated via Michael addition chemistry that incorporated an antimicrobial cationic block copolymer of PEG and polycarbonate. The antimicrobial mechanism of the cationic hydrogels is proposed that the anionic cell wall/membrane of bacteria was first attracted and interacted with the cationic hydrogel surface at many fixed points via electrostatic interaction, followed by the insertion of the hydrophobic segments of hydrogel into the hydrophobic regions of the lipid membrane, inducing the leakage of the membrane and eventually resulted in cell lysis. These hydrogels were then grafted onto silicone rubber, a material used to manufacture catheters. The antimicrobial activity of hydrogel-coated rubber was investigated by exposing itself to *S. aureus* for 1 day. There were numerous viable *S. aureus* cells found on the rubber surface without coating, but no cells on the gel coated rubber surface were detected by confocal. No adverse effect of the gel was observed on the toxicity, skin sensitization and skin irritation [34].

**Fig. 4:**
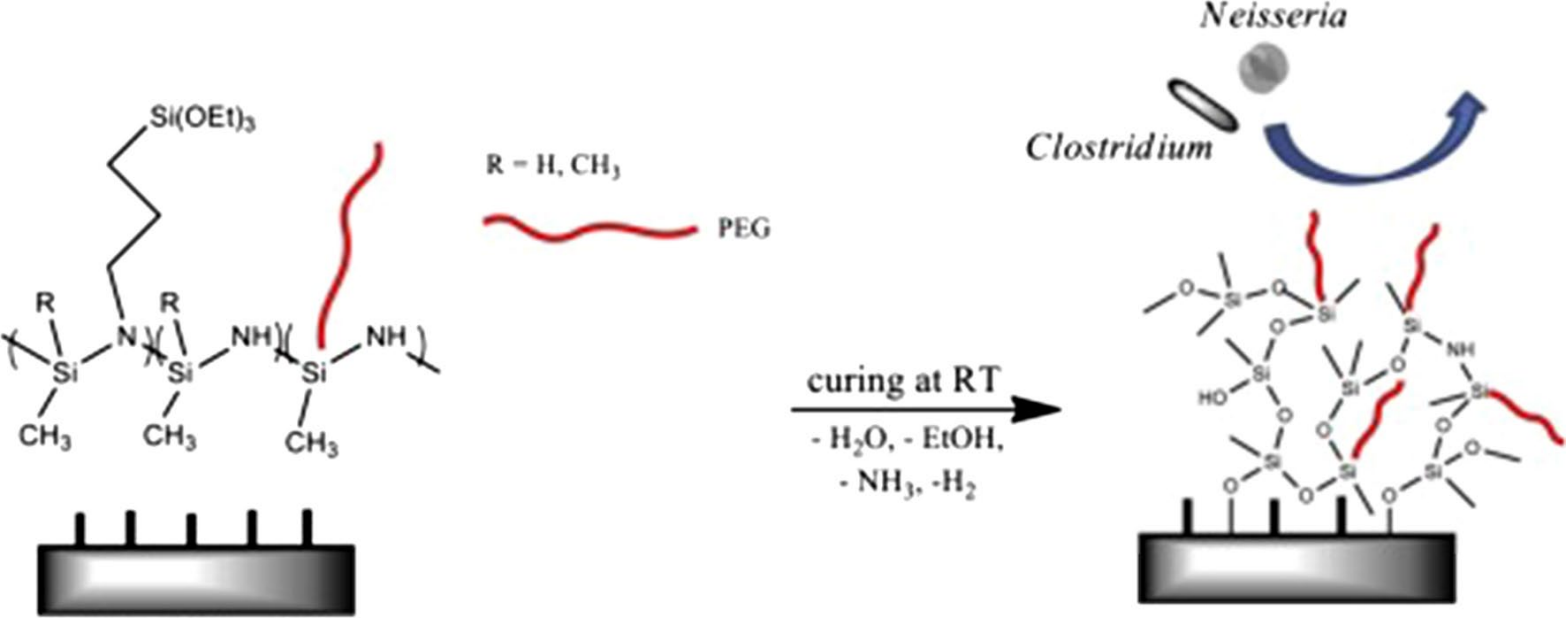
Silazane networks grafted with PEG branches for the marine biofouling anti-adhesion [39] reproduced with permission from Elsevier

PEG chains are also used to develop coating materials to prevent marine biofouling on a surface [35–39]. Nguyen et al. constructed new antifouling coatings by covalent grafting of methoxy-terminated PEG chains onto polysilazane polymers through a highly stable silicon carbon bond using a hydrosilylation reaction (Fig. 4) [40]. The resistance of the modified PEG materials to the adhesion of three marine bacteria species, bacillus *clostridium* sp. *SR1*, micrococcus *Neisseria* sp. *LC1,* and micrococcus *Neisseria* sp. *SC1* was investigated. The grafting of PEG chains onto polysilazane exhibits lower levels of bacteria adhesion. Their further study demonstrated that the surface density of the PEG chains has a greater effect in inhibiting bacterial adhesion. With the increase in PEG graft density, the ability of this antifouling coating to inhibit bacterial adhesion increases. There is insufficient demonstration of long-term stability of antifouling coatings in Nguyen’s article. However, the long-term fouling resistance of PEG to marine fouler was tested in Gruze and colleagues’ study [41]. It was shown that PEG was oxidized in seawater and lost its antifouling properties with time and was then settled much like any other surface submersed in the spore solution.

### Stimuli-responsive polymers

Stimuli-responsive, or “smart” polymers, have the extraordinary ability to change their physical and chemical state after they “detect” a change in their environments; their responses depend dramatically on their chemical composition [42]. In addition, due to the ease of modification with specific chemical functionalities, stimuli-responsive polymers have attracted additional attention for use as antifouling coatings [43–46]. Some researchers have used the properties of stimuli-responsive conformational changes to develop antifouling coating materials with self-cleaning properties [47, 48]. Compared to traditional antifouling surfaces, which are often associated with the accumulation of dead bacteria and other debris that degrade biocidal activity and provide nutrients for other colonizers [49], the stimuli-responsive polymer-modified surface is desirable for foulants to be removed or released to maintain long-term anti-fouling properties.

**Fig. 5:**
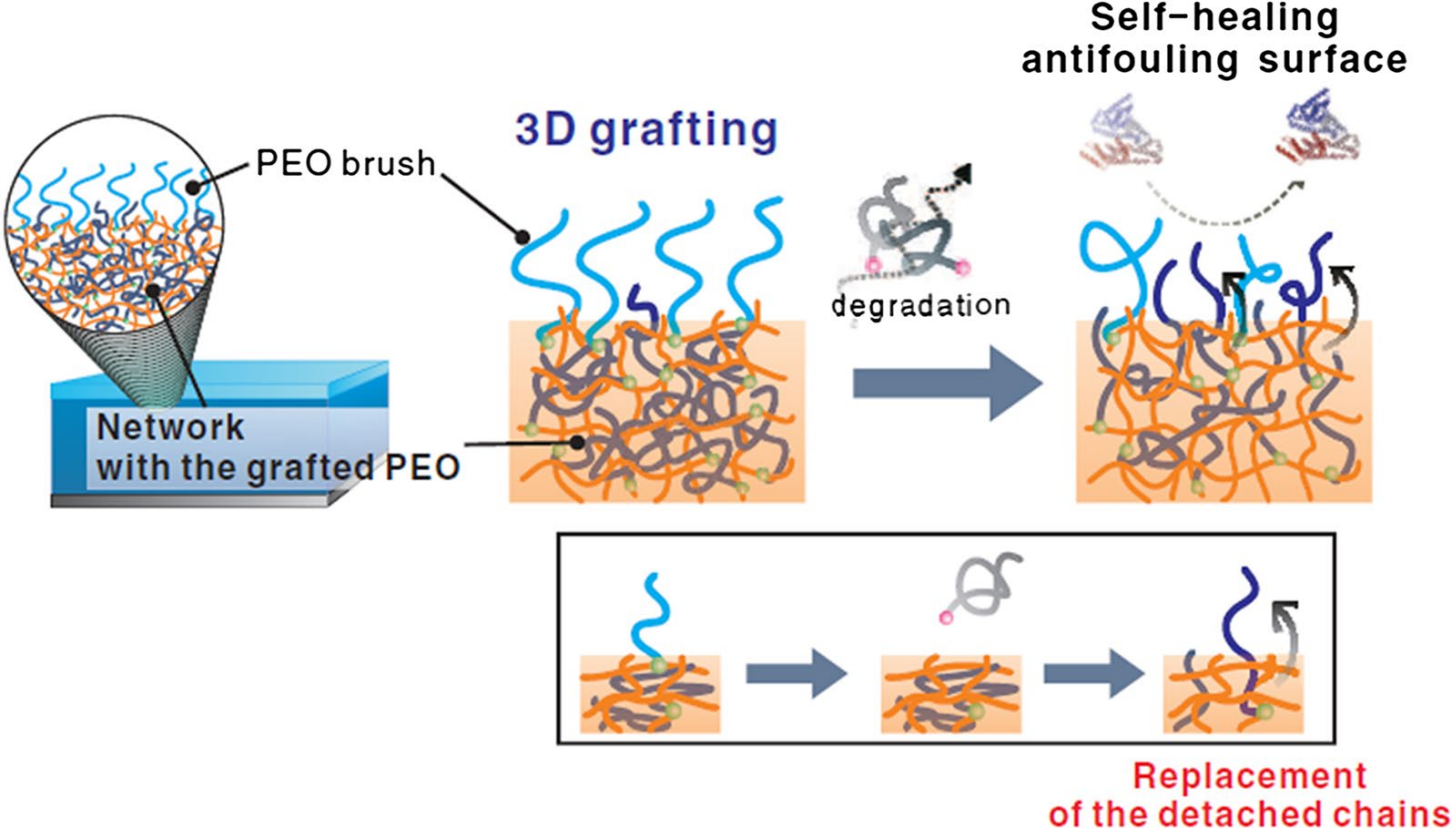
PEO grafting on the surface and inside of a P2VP film. The self-healing aspect of the antifouling property is due to the rearrangement of internally grafted polymers to the interface (marked as *dark blue* chains) [48] reproduced with permission from WILEY–VCH Verlag GmbH & Co

A novel stimuli-responsive material is reported to conquer the degradation and detachment of the grafted antifouling molecules, which can lead to a loss of the antifouling effect. In this study, pH-responsive cross-linked poly(2-vinyl pyridine) (P2VP) films (10–20 nm in thickness) on the surface of Si-wafer were prepared first. Poly(ethylene oxide) (PEO) was next grafted to the surface and inside the P2VP network films. Because P2VP and PEO are miscible, PEO chains can penetrate into the P2VP network. The network of P2VP acts as a reservoir of PEO chains. Because P2VP film has pH-responsive properties (swell and collapse if the pH value is varied) [50], the prepared polymer network can replenish the lost PEO chains at the network interface (Fig. 5). The rearrangement of polymeric chains due to pH change provides the basis for the self-healing effect. The antifouling properties of the PEO-grafted films were studied by protein adsorption tests using bovine serum albumin (BSA) and fibrinogen. The results demonstrated that the adsorbed amounts of protein on the PEO-grafted films were negligibly small, and this pH-responsive poly(2-vinylpyridine) films with the 3D grafting of poly(ethylene oxide) demonstrate a fourfold increase in longevity of antifouling behavior [51].

**Fig. 6:**
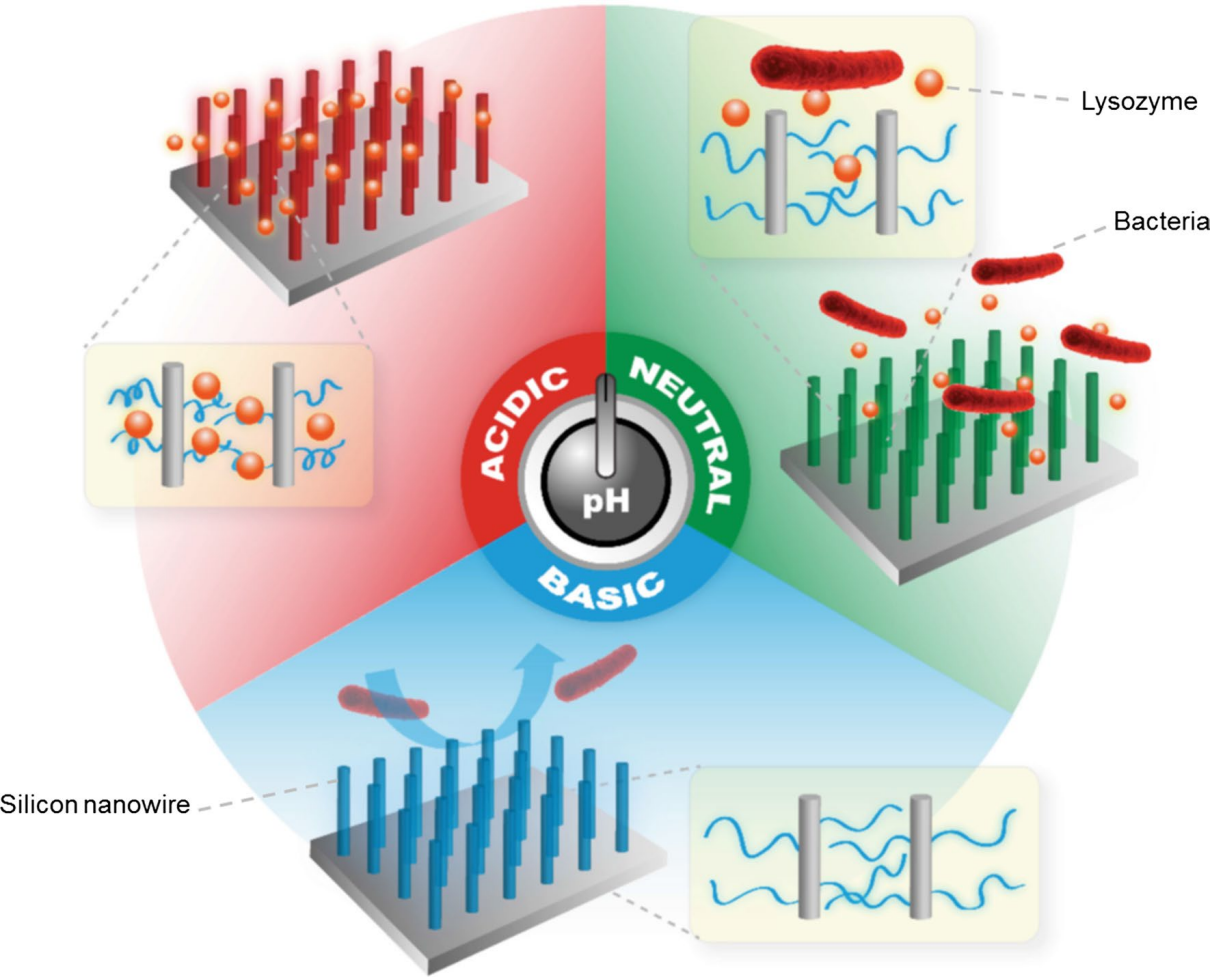
Schematic illustration of a smart antibacterial surface with pH-responsive capability of loading biocide, killing bacteria, and releasing bacteria [50] reproduced with permission from WILEY–VCH Verlag GmbH & Co

In another study, vertical silicon nanowire (SiN) arrays with uniform lengths and densities have been prepared as a substrate. SiN nanoscale structure has a porous structure and high-surface-to-volume ratio. Because of those advantages in structure, SiN can serve as a good reservoir with a high loading capacity for biocidal agents, and therefore was considered in this study. After the preparation of SiN nanowire arrays through a reported chemical etching method [52], the nanoscale SiN wires were then modified with a pH-responsive polymer, poly(methacrylic acid) (SiN-PMAA). Because SiN-PMAA surfaces exhibit pH-responsive protein adsorption behavior under acidic conditions, Lysozyme, an antibacterial enzyme, was adsorbed on the SiN-PMAA surfaces for use in biocidal applications. It was hypothesized that lysozymes bind to SiN-PMAA surfaces through hydrogen bonds formed between –COOH groups on PMAA chains and the –CONH– groups on proteins. When the condition becomes neutral, the –COOH groups on PMAA chains are ionized into –COO^−^ groups, leading to the breaking of hydrogen bonds and thus reducing the interaction between the proteins and the surfaces. The loaded lysozymes are therefore released to kill the *E. coli* attached on the surface and suspended in solution near the surface. As the pH increases from 7 to 10, the ionization of –COOH increases resulting in increased negative charge density and farther extension of the polymer chains away from the surface. Those changes in the surface properties lead to the release of the attached dead bacteria from the surface (Fig. 6) [53].

We found that few research papers have reported on the use of stimuli-responsive polymers modified silicon and silicon-based materials for marine applications. Therefore, more research in this area is expected in the near future. An intrinsic difficulty of these stimuli-responsive polymers-coatings is the trade-off between the ability to switch and the mechanical stability of the system. At low crosslink densities, the switching process works well, but the coatings are quite fragile. At high crosslink densities, the coatings are more robust, but switching becomes increasingly difficult [54].

Incorporation of biocidal agents, such as synthetic biocides and enzymatic biocides, on surfaces is an effective approach to kill or degrade attached bacteria, and therefore inhibit their proliferation and formation of biofilms. For example, quaternary ammonium salt (QAS), one type of synthetic biocide, can provide effective protection against bacterial colonization by disrupting the cell membrane through the binding of their ammonium cations to anionic sites in the outer layer tissue of bacteria [21]. It was reported that the QAS-modified substrate can resist the bacterial adhesion with water-repelling hydrophobicity and eradicate the contacted bacteria with biocidal capability [55].

**Fig. 7:**
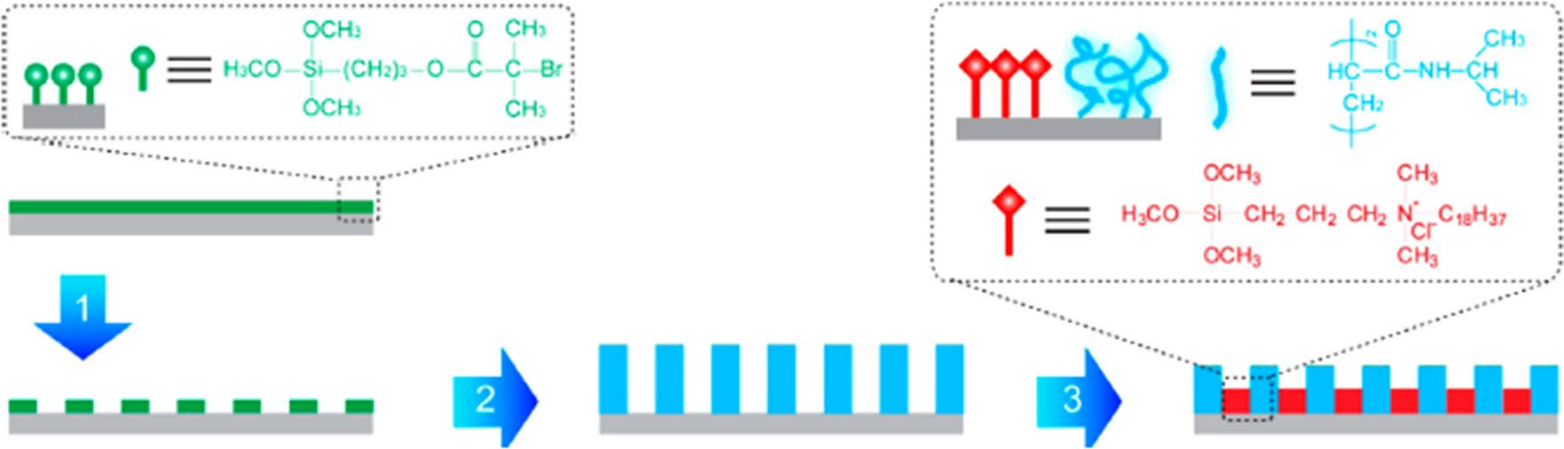
Schematic depiction of the procedure for the preparation of nanopatterned PNIPAAm surfaces (Steps 1 and 2) and nanopatterned PNIPAAm/QAS Surfaces (Steps 1–3) [60] reproduced with permission from Royal Society of Chemistry

**Fig. 8:**
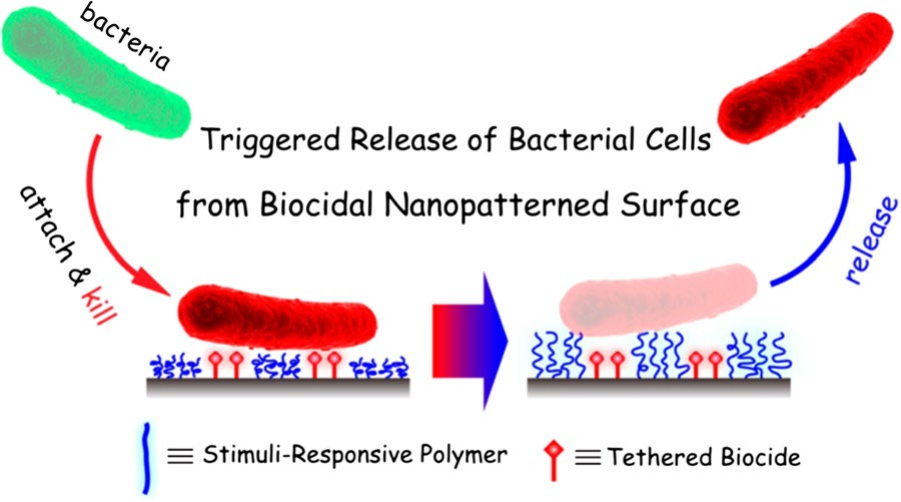
Upon a reduction of the temperature, swollen PNIPAAm chains promote the release of dead bacteria [60] reproduced with permission from Royal Society of Chemistry

The López lab at Duke University combined the antimicrobial activity of QAS and stimuli-responsive polymers, poly(*N*-isopropylacrylamide) (PNIPAAm), which have been previously shown to controllably release adsorbed organisms [56–60], resulting in effective killing and continued de-attachment of dead bacteria [61]. First, the silicon wafer was cleaned with “Piranha” solution. This process results in the formation of a thin silicon dioxide layer. Then, self-assembled monolayers (SAMs), terminated with atom transfer radical polymerization initiators, was immobilized on the wafer. Interferometric lithography (IL) was then used for the surface patterning. PNIPAAm polymer brushes were then grafted onto the patterned SAMs using surface-initiated activators regenerated by electron transfer-atom transfer radical polymerization. The nanopatterned PNIPAAm surfaces were then incubated in QAS solution to produce hybrid surfaces (QAS was integrated onto the substrate between nanopatterned PNIPAAm brushes, Fig. 7). The prepared surface was tested against *E. coli* K12. The bacteria were killed as they were exposed to QAS moieties. Furthermore, the reduction of the temperature results in the swollen PNIPAAm chain which promotes the release of dead bacteria (Fig. 8). In their later study, lysozyme replaced QAS and served as a biocide. The surface exhibited the same ability to control the attachment, killing and release of bacteria in response to temperature changes. Compare to QAS, lysozyme is environmentally friendly, requires no toxic precursors or cost disposal protocols [62].

### Zwitterionic polymers

Zwitterionic polymers are polymers that have moieties possessing both cationic and anionic groups. These materials are characterized by high dipole moments and highly charged groups but are charge neutral [63]. The strongly bound hydration layer, induced by electrostatically ionic solvation in addition to hydrogen-bonding interactions, is considered to be the reason for the efficient repulsion of fouling materials—the electrostatic interactions between water molecules and dipoles present in the zwitterionic polymer chains make these polymers better “water-bears” [18, 64, 65]. For example, Huang’s group recently modified the silicon-based materials, PDMS, with sulfobetaine silane (SBSi) with covalent silanization. This superhydrophilic zwitterionic interface presents its antibacterial adhesion property to resist nonspecific adsorption of bacteria (*S. epidermidis* and *P. aeruginosa*), protein (bovine serum albumin, lysozyme, and mucin), and lipids. Moreover, because the cellular liability experiment demonstrated that SBSi had negligible cytotoxicity in vivo application, the applicability of SBSi modification was applied to silicone hydrogel contact lenses by following the same procedure as that for PDMS. The SBSi-modified contact lens was kept under the *P. aeruginosa* solution in a physiological condition. The experimental results demonstrated that the number of adherent bacteria on SBSi-modified contact lens is much less than unmodified one [5].

Most zwitterionic polymers with antifouling functionality are attached to the surface through Si–O–Si–C [66–68] and Si–O–C linkages [69, 70]. A major disadvantage of these approaches is the limited hydrolytic stability. This may result in the detachment of the zwitterionic polymers and may consequently keep long-term application out of reach. To increase the stability of the attachment, researchers from Netherlands deposited the Si substrates with Si_x_N_4_ (x > 3) by low-pressure chemical vapor deposition (LPCVD) with a thickness of 150 nm first. The sulfobetaine methacrylate (SBMA) zwitterionic polymer brushes were then grafted from Si_x_N_4_ surfaces by controlled surface-initiated atom-transfer radical polymerization (ATRP) through more stable Si–C linkage as compared to less stable Si–O–Si–C and Si–O–C linkages. As a result, the long-term protein-repellent properties of the zwitterionic polymer (polySBMA) remain largely unaffected [71].

**Fig. 9:**
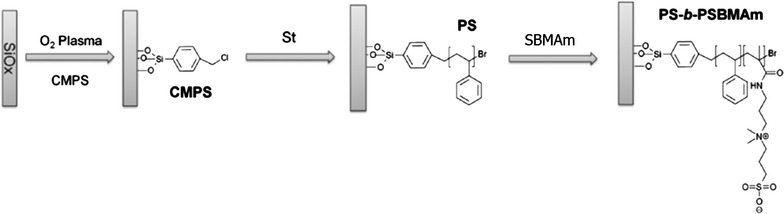
Synthesis of polymethyl methacrylate (PMMA) and polystyrene (PS) with two hydrophobic polymer blocks between PMMA and PS (PS-*b*-PSBMAm) on SiO_x_ surface via surface-initiated atom-transfer radical polymerization. CMPS refers to (p-Chloromethyl)phenyl-trichlorosilane), and SBMAm indicates sulfobetaine methacrylamide [73] reproduced with permission from ACS Publications

**Fig. 10:**
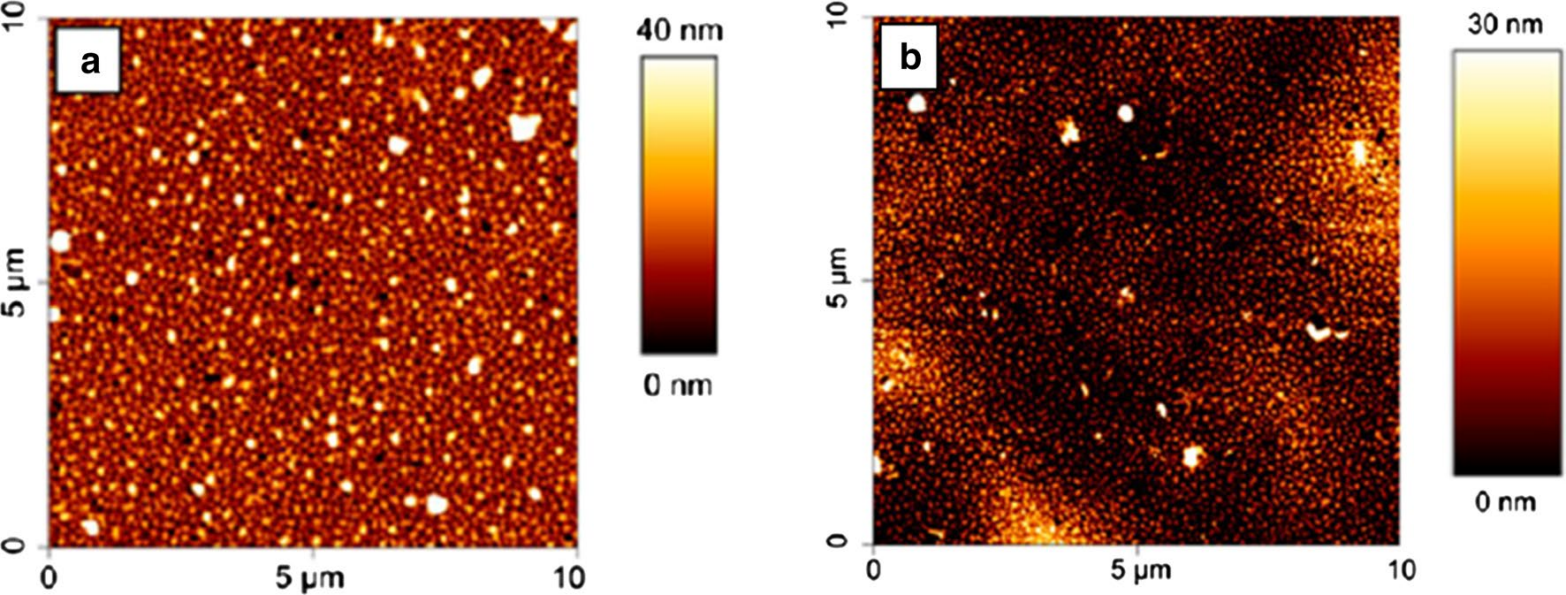
Tapping-mode AFM images in air of PS-b-PSBMAm block copolymer brushes before (**a**) and after (**b**) 4 weeks of immersion in seawater [73] reproduced with permission from ACS Publications

Due to the challenges in surface stability and corrosion, zwitterionic polymers have limited use in marine applications [72, 73]. To prevent cleavage of the anchoring segment and increase the long-term stability of zwitterionic polymer-based brushes in seawater, Vancso and co-workers investigate diblock copolymer-type brushes composed of bottom hydrophobic segments and a polysulfobetaine top on the substrate of SiO_x_ (Fig. 9) [74]. The hydrophobic nature of the protecting block limits water penetration into the brush; therefore, the stability of hydrophilic brushes in aqueous media grown from SiO_x_ substrates are enhanced [75]. The visual appearance of diblock architecture protected zwitterionic brushes did not change after long exposure to seawater (3 months). No sign of microbial growth was observed during the experiment (14 weeks). Atomic Force Microscopy (AFM) topographical measurements were used to measure the surface modified with the diblock copolymer brushes after 4 weeks of immersion, showing almost no differences in surface morphology (Fig. 10).

## Fabrication of surfaces with nano- or micro- topographical features

It is well-known that the surface microstructure influences cell behavior or tissue formation [76], and certain topography features may contribute to a fouling-free surface. In Carman’s study, the designated surface microstructures, including ridges and channels, were induced on a silicone elastomer based cross-linked PDMS films by adopting microfabrication techniques. Their results demonstrated that the settlement of *Ulva linza* zoospores related to the ridge topographies, and is inversely proportional to the width (between 5 and 20 μm) of the channels [77].

Because the special surface topography of skin or shells includes micro- and nanostructure, many marine organisms do not have biofouling [78, 79]; therefore, artificial surfaces with biomimicking natural microtextures have been fabricated and studied. In a recent work, researchers designed PDMS hierarchical surface microtopographies that mimic the critical features observed on the *M. hardwickii* surface. This micropatterned surface was subject to fouling tests, including laboratory assays against algae adhesion and was also exposed to marine environments during field testing. Their results demonstrated that the settlement of organism on the patterned PDMS was lower than that on the smooth PDMS, indicating that the designed micrographic surface features associate with antifouling. The micropatterned PDMS samples were further modified with zwitterionic polymer brushes, and it was reported that the use of microtopography enhances the antifouling performance of zwitterionic polymer brushes to a greater extent [80].

Another pioneering report of biomimicry focused on the surface of the *Trifolium* leaf, which has a self-cleaning property. In this work, silicone elastomer was used to fabricate biomimetic surfaces using the *Trifolium* leaf as a template. The surface of the replica displays a remarkable amount of microspines with a size similar to that of the original *Trifolium* leaf, and is effective in resisting the settlement of microalgae. The antifouling property of the replica was improved by modification with poly(3-sulfopropyl methacrylate) (PSPMA), a kind of hydrophilic acrylate polymers [81].

**Fig. 11:**
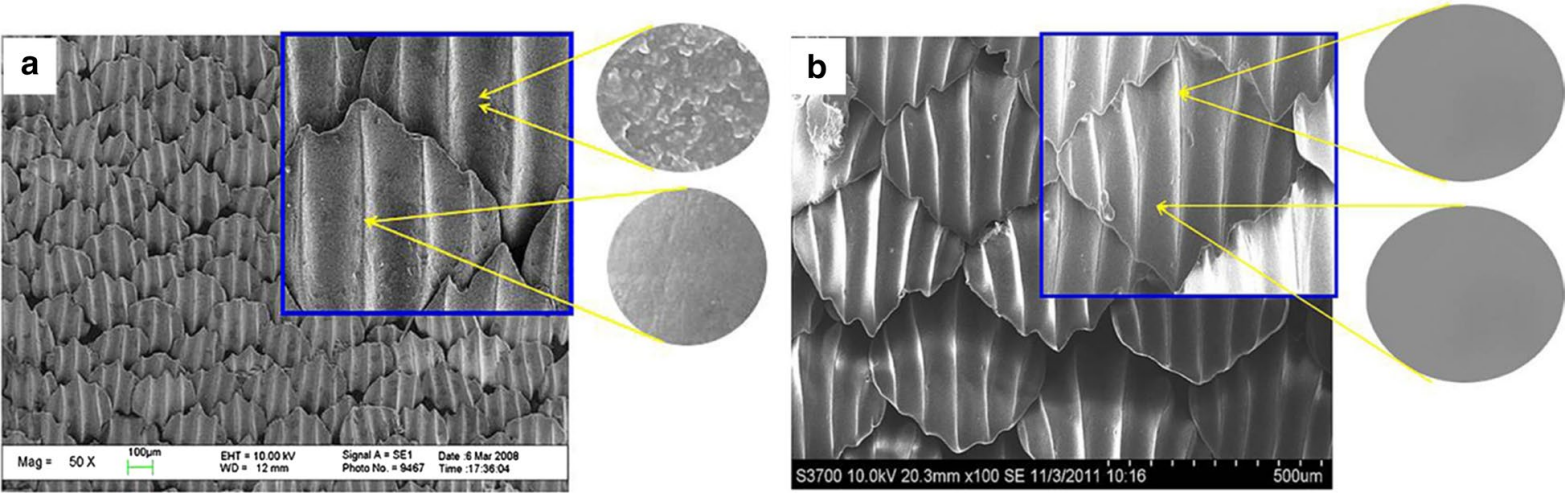
Scanning electron microscopy (SEM) images of shark skin surface (**a**) and the surface of biomimetic shark skin (**b**). The high-magnification SEM images of shark skin surface show that the ridge possesses relatively smooth surface structure. However, some nanostructured protuberances were found on the concave groove surface. The high-magnification SEM images of biomimetic shark skin demonstrate a very smooth surface which does not contain any nanostructured protuberances [79] reproduced with permission from Biology Open

In a recent study, Huang et al. [82] fabricated biomimetic surfaces of shark skin onto the surface of PDMS (Fig. 11). BSA and ovalbumin (OVA) were used as a model for protein and glycoprotein to study the surface’s anti-protein adhesion properties. The contact angle variation after the surface soaked in protein solution indicates that the fabricated biomimetic surface microstructure can block the adhesion of protein.

## Conclusion and perspectives

We have reviewed strategies for designing effective antifouling approaches for silicon and silicon-based materials, although several of them have associated shortcomings. In addition, by providing a surface topography that is unfavorable for biofoulant attachment, it can also repel the attached biofoulant from the silicon and silicon-based material surface. Although many works claim their antifouling coatings or surface modifications have long-term stability, it is our understanding that an antifouling coating doesn’t last forever; as it becomes aged, it becomes less effective. It also has been brought to our attention that once the deposition of foulants has taken place, the surface modification is no longer effective at preventing fouling, which is understandable in light of the fact that the effect of solute/coating interaction is severely reduced once a layer of deposited foulants is formed [83]. In other words, the development of an absolutely nonfouling surface is extremely difficult. The old antifouling coating needs to be removed, and a new antifouling coating needs to be applied once the fouling layer is formed. One of the approaches to remove antifouling paint is by scraping, which is a time consuming process. In addition, one might damage the surface during this coating removal process. We must therefore explore methods by which to restore the permanently fouled surface and maximize the effective use of the modified materials. We expect that these methods can be described in near future and become solutions to reduce the cost associated with fouling for industry, and can prevent long-term bio-fouling for those biomedical devices which are fouled over quickly, such as the colonization of bacteria on catheters, contact lenses, and surgical tools, so that the healthcare costs can be decreased. Besides, we expect more studies testing the cost-effectiveness, durability, and stability of those designs in real situations, such as the prevention of marine fouling and fouling on medical implants/devices. However, in those cases, the challenge would be developing a coating or an approach of modification that will resist adhesion of all forms of biofouling.

## Abbreviations

CAUTI: Catheter-associated urinary tract infection
PDMS: polydimethylsiloxane
PEG: poly (ethylene glycol)
EO: ethylene oxide (EO)
HAS: human serum albumin (HSA)
Lys: lysozyme (Lys)
P2VP: poly(2-vinyl pyridine)
PEO: poly(ethylene oxide)
BSA: bovine serum albumin
SiN: silicon nanowire
PMAA: poly(methacrylic acid)
QAS: quaternary ammonium salt
SAMs: self-assembled monolayers
IL: interferometric lithography
PNIPAAm: poly(N-isopropylacrylamide)
SAMs: self-assembled monolayers
SBSi: sulfobetaine silane
AFM: Atomic Force Microscopy
LPCVD: low-pressure chemical vapor deposition
PSPMA: poly(3-sulfopropyl methacrylate)
OVA: ovalbumin
SEM: Scanning Electron Microscopy
